# An all-acoustic toolbox for performing unit operations and their combinations on levitated droplets

**DOI:** 10.1016/j.ultsonch.2025.107611

**Published:** 2025-10-11

**Authors:** Jianqing Li, Nicholas J. Goddard, Khalid Haliru, Ruchi Gupta

**Affiliations:** School of Chemistry, University of Birmingham, Birmingham B15 2TT, UK

## Abstract

Automatic and contactless manipulation of droplets in mid-air has wide potential applications from materials processing to biochemistry and pharmaceutical development. To perform (bio)chemical reactions in a contactless and automated fashion, herein, we report an all-acoustic toolbox for performing unit operations (generate, hold, move and merge) on droplets with integrated optical detection. The toolbox was realized using phased arrays of ultrasound transducers in combination with pump and capillary nozzles to generate pendant droplets of required volumes and pulling them away from the tip of the nozzles using acoustic forces, then using the acoustic forces to move and merge droplets followed by quantitative measurements using the integrated optical detection. This is an unprecedented report on droplet generation by pulling pendant droplets using acoustic forces. The generation of aqueous droplets containing different percentages of dimethyl sulfoxide was possible up to 50 % DMSO. The error in the volume of aqueous droplets generated using the approach was between 5.6 and 13 % depending on the volume of the droplet and the transducer voltage. The success rate for holding droplets varied between 78 and 95 % depending on the droplet size and transducer peak-to-peak voltage. Up to six droplets in two columns of three could be generated, moved and merged. Subsequently, a sequence of unit operations was created to implement an esterase enzyme assay, which is widely used in biochemical labs and industrial processes, in levitated droplets. The reaction rate was higher in levitated droplets (2.17 to 5.21 × 10^−3^ s^−1^ compared to that in microtiter plates (∼2.0 × 10^−3^ s^−1^), which we hypothesize is a result of stirring of the droplet by acoustic streaming. Future work will focus on increasing the number of droplets that can be levitated and manipulated using our all-acoustic toolbox. The adoption of our all-acoustic toolbox for performing (bio)chemical reactions will not only increase automation but also reduce the use of single-use non-recyclable plastics such as microtiter plates and pipette tips.

## 1 Introduction

We present an all-acoustic toolbox for performing unit operations (generate, hold, move and merge) and combinations of these unit operations on much larger numbers of levitated droplets than previously reported. Our all-acoustic toolbox is integrated with optical detection for quantitative measurements. Equally, we present a novel method for generation of levitated droplets by pulling pendant droplets at the end of capillary nozzles using acoustic forces. Acoustic traps were created at the ends of nozzles and then moved away to pull the pendant droplets for generating levitated droplets. The process was repeated to generate and hold columns of levitated droplets. Two columns of levitated droplets were then moved towards each other and eventually merged using acoustic forces as described in our 2025 publication [1]. While the generate operation allowed automated dispensing of reagents of (bio)chemical reactions, the move and merge operations allowed the reagents to be brought together to start (bio)chemical reactions. The progression of (bio)chemical reactions in levitated droplets were monitored using an integrated optical detection set-up. Furthermore, we present the utility of our all-acoustic toolbox for performing a (bio)chemical reaction, esterase enzyme assay, in a contactless and automated fashion. Automated (bio)chemical reactions have applications in various disciplines including biology [2,3], chemistry [4,5] and pharmacy [6]. The adoption of our all-acoustic toolbox will not only increase automation but also reduce the colossal amount of plastic such as pipettes and microtiter plates used in labs, currently ∼7 million tons every year worldwide [7,8].

Before this work, only two reports have shown acoustic systems to automatically generate, move and merge levitated droplets. The acoustic systems in both these reports comprised a piezoelectric droplet generator integrated with a 16 × 16 phased array transducer (PAT) and a reflector with holes for generating ultrasound waves and for injecting/ejecting droplets in/out, respectively. Andrade et al. [9] generated a droplet, moved it away from the droplet generator and then generated another droplet of the same liquid. Subsequently, the two levitated droplets were moved and merged by modulating the phases of the PAT. The droplets were discarded by turning off the voltage used to drive the PAT. Chu et al. [10] showed sequential generation of up to 6 droplets. The generation of each levitated droplet was followed by move and then dropping each droplet in a mixing well in a solid surface. This implies that the system reported by Chu et al. [10] was not completely contactless because the mixing of droplets was performed in a container. Although the acoustic system reported by Andrade et al. [9] allowed contactless manipulation of droplets, droplets could only be moved in the plane parallel to the reflector and only up to two levitated droplets were manipulated simultaneously. In 2025, authors reported an acoustic system with 16 × 16 PATs forming top and bottom faces as well as a novel DS-PAT algorithm to increase the number of acoustically levitated droplets to 12 [1]. Additionally, up to 6 pairs of droplets were merged simultaneously and up to 4 merge operations were performed in-sequence. However, in the 2025 report by the authors and other previous studies by the authors [4,11] and others [[12], [13], [14]] on acoustic levitation and manipulation, droplets were inserted in acoustic traps manually. The manual introduction of droplets in acoustic levitators require operators to carefully locate the target levitation points and avoid sample losses, resulting in low efficiency and reproducibility because of human fatigue and errors.

Acoustic droplet ejection (ADE) was reported for the first time in 1920 s [15] and many review articles have been reported [[15], [16], [17]]. There are a few ADE methods to eject droplets from fluid reservoirs [9,10,[18], [19], [20]]. In one of the ADE methods, ultrasound signal emitted by transducer(s) is focused and travels through an ultrasound matching liquid to a reservoir filled with liquid (reagents, hydrogel containing cells) to generate a droplet. High-intensity ultrasound is focused to deform the liquid–air interface and overcome surface tension, propelling a liquid droplet in air. The volume of ejected droplets can be tuned by changing the frequency of ultrasound. For example, 4.6 to 13.6 MHz ultrasound allows generation of 50–200 µm diameter, which corresponds to <5 nL volume droplets. This method is beneficial for the generation of droplets containing cells because cells experience minimal shear stress. The ejected droplets can be deposited on a movable building platform to make 3D cell systems. Another advantage of this method is that droplets are ejected directly from the reservoir, eliminating the need for narrow and difficult to clean fluidic conduits [19]. However, ADE has so far only been used to generate <5 nL volume droplets. To generate larger volume droplets, nozzles and ultrasound transducers are used. For example, Daniel et al. generated 0.5–1.4 mm diameter or 65–1400 nL volume 20 cS silicone droplets using nozzles and ultrasound transducers. The variation in the diameter of droplets generated using this droplet generation approach was ±1 % and a series of droplets could be generated sequentially by applying 25–30 V pulses with a width of 400–500 µs to the transducers [18], but different diameter nozzles were required to change the volume of generated droplets. Furthermore, the report by Daniel et al. did not demonstrate the generation of droplets of water and organic solvents (e.g., dimethyl sulfoxide) that are commonly used in which to perform (bio)chemical reactions.

Herein, we report a unique all-acoustic toolbox to perform unit operations (generate, hold, merge and move) and any combination of the unit operations on levitated droplets for performing (bio)chemical reactions. Equally, we report a novel droplet generation approach where a syringe pump was used to create a pendant droplet of selected volume (typically, 2–3 µL) at the end of a capillary nozzle following which the pendant droplet was pulled using acoustic forces by PATs. Our method offers distinct advantages over acoustic droplet ejection (ADE) by enabling convenient and accurate control over droplet volume regardless of liquid properties, along with the ability to generate larger droplets at the µL scale. Unlike ADE—which requires repeated adjustment of pulse parameters to compensate for variations in liquid height and properties—our system uses separate pumps and nozzles to accurately and conveniently ensure volumetric consistency. Furthermore, whereas ADE must overcome both capillary and gravitational forces to eject nL-scale droplets, our approach leverages gravity and acoustic force together to readily detach larger droplets (∼2–3 µL) from nozzles. The process was repeated to generate columns of droplets, which were moved and merged to start (bio)chemical reactions in levitated droplets. A sequence of the unit operations was then performed to implement esterase enzyme assay in levitated droplets while obtaining quantitative data using the integrated optical detection set-up. The reported toolbox is a big step towards fully automated and contactless handling of droplets for performing (bio)chemical reactions.

## 2 Experimental

### 2.1 Materials

Amaranth (A1016), dimethyl sulfoxide (DMSO) fluorescein diacetate (FDA, F7378), and porcine liver esterase enzyme (E2884) were purchased from Sigma Aldrich Ltd (Gillingham, Dorset, UK). Phosphate-buffered saline (PBS, 10X), pH 7.4 was purchased from Thermo Fisher (Loughborough, UK). Other materials used in this work including their suppliers are as follows: glass capillary with internal and external diameters of 0.3 mm and 0.5 mm, respectively (Amazon, UK), 160 mm long 20 G stainless steel tubing (Stainless Tube and Needle Company, Tamworth, UK), PVC tubing with 0.76 mm internal diameter (Gilson™ F117936, Fisher Scientific, Loughborough, UK), 1 mL syringes with luer slip tip (Z683531, Sigma Aldrich, Gillingham, Dorset, UK), ferrules (Cole-Parmer nylon female luer to hose barb, 12664615, Fisher Scientific, Loughborough, UK).

### 2.2 Instrumentation

**Fig. 1:**
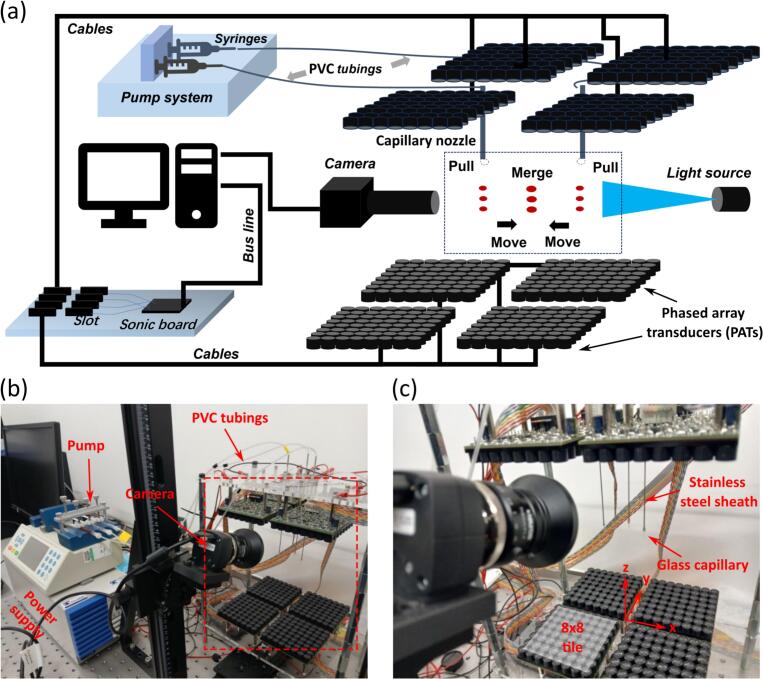
(a) An illustration and (b) experimental set up of the all-acoustic toolbox, and (c) shows a magnified view of the PATs and capillary nozzles used for droplet generation where the top and bottom PATs are separated by 173 mm.

The experimental set-up is shown in Fig. 1 where the PATs are similar to that described by the authors in [1,11]. Briefly, ultrasonic transducers (MA40S4S, DigiKey Inc., Minnesota, USA) of 10 mm diameter that produced sound waves of 40 kHz were used to form PATs. Each 16 × 16 PAT, which formed the top and bottom face of the acoustic levitator, was made of 4 tiles of 8 × 8 transducers as shown in Fig. 1(c). As shown in Fig. 1(a) and (b), PATs were driven by a power supply (Kikusui PWR801L, Telonic Instruments, Wokingham, UK). Unless stated otherwise, 16 V peak-to-peak was used. The phases of transducers were controlled by a program written in-house using C++ Builder (Embarcadero Inc, Texas, USA). A csv file containing the phases and peak-to-peak voltage was loaded to the in-house written program. The phase and voltage information was sent to the two-side 16 × 16 arrays. After loading all the phase files including pull, move and merge, the program sent the phases step by step with an interval of several seconds. There was no real-time adjustment to the phase during the whole process as most of our operations including pull, move and merge of droplets were sufficiently stable.

The 8 × 8 tiles were mounted on 5 mm thick PMMA sheet with the edges of the neighboring tiles separated by 10 mm (one transducer diameter). In the gap separating the neighboring tiles, there was a grid of holes of diameter 5 mm and a pitch of 20 mm through which nozzles were inserted. Each nozzle was made of a glass capillary threaded through a stainless steel tube leaving 5 mm of the glass capillary exposed and fixed in place using cyanoacrylate adhesive (see Fig. 1(c)). Each glass capillary and stainless steel assembly was in turn mounted in a 3D printed fixture that was screwed onto the PMMA sheet. As shown in Fig. 1(a) and (b), each stainless steel tube was connected to a 1 mL syringe using a combination of fluidic ferrule and PVC tubing. The syringes were mounted on a syringe pump (Chemyx Fusion 400 Syringe Pump, Chemyx, Texas, USA). The start/ stop time and flow rate of the syringe pump was controlled by an additional subtroutine in the PAT control program to ensure that the operation of the pump was synchronized with changes to the PAT phases.

A daylight wafer light box (D/E/U/A 35040, 4 W LEDs, Amazon, UK) was used as a light source to study unit operations and sequences of unit operations on droplets. A high-power blue LED (Luxeon L135-B475003500000, RS Components, Corby, UK) with a peak wavelength between 469 and 480 nm was used to illuminate the droplets for studies on enzyme assays. Monochrome images were recorded using a Daheng Imaging MER2000-19U3M USB3 camera (GeT Camera BV, Eindhoven, Netherlands) with 5496 × 3672 pixels resolution equipped with a Hayear HY-300XA zoom lens (Shenzhen Hayear Electronics, Shenzhen, China) with magnification 0.7 to 4.5×. For recording fluorescence images, a 520 nm interference filter with 10 nm bandpass (Knight Optical, Harrietsham, UK) was placed in front of the zoom lens. Images from the Daheng camera were recorded at selected intervals synchronized to the PAT control program. Movies were recorded using a color camera (UI3580LE-C-HQ USB3, IDS Imaging Development Systems, Obersulm, Germany) with 2560 × 1920 pixels resolution equipped with a widefield imaging 25 mm focal length lens (MVL25M23, Thorlabs Inc., USA). Another color camera (MER2-2000-19U3C, China) with 5496 × 3672 pixels resolution equipped with a widefield imaging 25 mm focal length lens (Navitar, New York, USA) was used to capture color images.

### 2.3 Software and algorithm

The PATs, syringe pump and stills cameras were controlled using a program written in-house using C++ Builder, which was described previously [1]. The speed at which liquids were dispensed from the syringe pump was set at 10 µL/ min.

To calculate the phases of transducers (φ_ij_), our DS-PAT algorithm [1] was used. The DS-PAT was adapted from the hologram design method [[21], [22], [23]] and allowed finding a global minimum for a defined objective function in the parameter space of transducer phases based on Metropolis criteria. Compared to traditional algorithms such as IBP [24] and Diff-PAT [25], DS-PAT is more likely to approach the global minimum. In this work, the objective function was C_2_(φ_ij_), which is defined in Equation (1).

(1) $$C_{2}\left(\varphi_{\mathrm{ij}}\right)=-\sum_{n}\left|p_{n}\right|+\alpha \cdot \sqrt{\frac{\sum_{n}{\left|\left|p_{n}\right|-\overset{¯}{\left|p_{n}\right|}\right|}^{2}}{N}}$$

where, |*p_n_*| is the average pressure and the second term on the right side of Equation (1) is the standard deviation of the pressure at each target point, *n*. *N* and |*p_n_*| with an overline are the total number of target points and calculated average pressure, respectively. *α* was tuned to control the magnitude of pressure at the target points. The possible phase of each transducer 0 to 2π was discretized into 64 levels, changing from 0 to 2π×(63/64). The initial phase of transducers was either a random value from 0 to 2π×(63/64) or set to 2π×(63/64). |*p_n_*| was 4000–5000 Pa and *α* was 25. The end criterion was defined as the percentage of transducers that changed phases in one iteration divided by the total number of transducers and was set to 0.05 %. The iterations required to satisfy the end criterion were typically around 60.

Image J was used for analysis of images and videos. ImageJ’s threshold and particle analysis routines were used to find the centroid as well as the major (*a*) and minor (*b*) axis of an ellipsoid fitted to each droplet. The levitated droplets were axisymmetric ellipsoid along the *z* axis and hence their volume was calculated using by Equation (2).

(2) $$V=\frac{4\pi }{3}{\left(\frac{a}{2}\right)}^{2}\left(\frac{b}{2}\right)$$

The camera calibration factor (pixel to distance) was determined using the center-to-center distance between the nozzles, which was 40 mm. For extracting grayscale values droplets, the center of each droplet was found, after which a box 100 pixels wide and 200 pixels high was drawn around the center of the droplet, and the mean and standard deviation of the grayscale values in this box was determined. This was done to avoid including reflections of the light source from the surface of the droplet.

## 3 Results and discussion

### 3.1 Unit operations

#### 3.1.1 Generate and hold

**Fig. 2:**
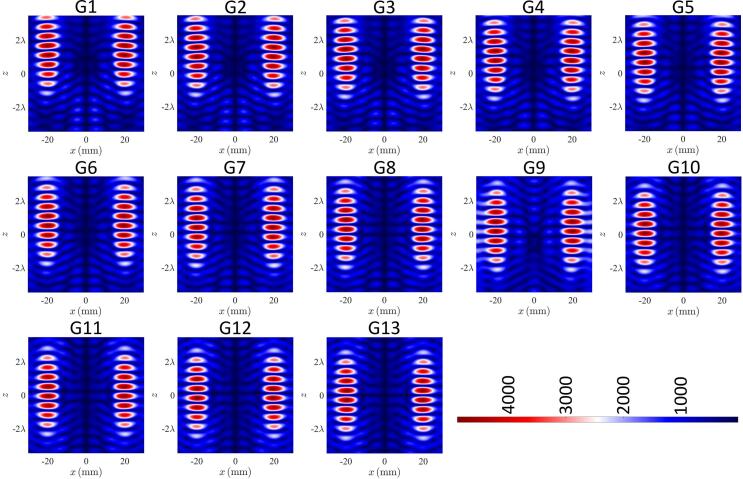
Simulated pressure distribution of the acoustic fields used to generate, position and hold 3 pairs of droplets where the 1st 2nd and 3rd pairs of pendant droplets were pulled using fields 2, 7 and 12, respectively while the remaining fields were used to position and hold droplets in mid-air.

**Fig. 3:**
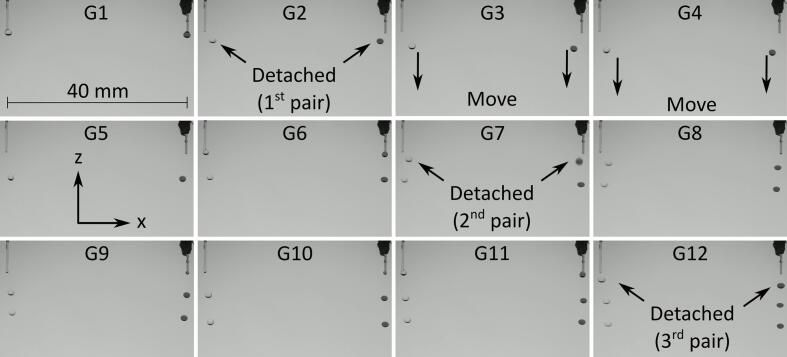
Images showing generation, positioning and holding of 3 pairs of droplets where the numbers 1–11 correspond to acoustic field numbers in Fig. 2, Fig. 2 µL solution was dispensed from the pump to create each pendant droplet, droplets on left are water and on right are amaranth in PBS.

The droplet generation comprised dispensing a selected volume of solution to create a pendant droplet at the tip of the capillary nozzle and then pulling the pendant droplet using acoustic forces. To generate and hold 3 pairs of levitated droplets, 13 different acoustic fields were used sequentially. The pressure distribution in each case is shown in Fig. 2. These acoustic fields were obtained by setting the *x* coordinates of the traps to be −20 mm and 20 mm that corresponded to the x coordinates of the central axis of the two capillary nozzles. To pull 3 pairs of droplets in sequence, 3 nodes were constructed along the z-axis at each x coordinate. To generate initial acoustic nodes, the z coordinates of the focal points were 19.5 mm, 14.5 mm, 9.5 mm and 4.5 mm. The distance between the neighboring focal points was set to 5 mm, which was ∼ half of the wavelength in air of sound waves emitted by the transducers. The 4 focal points at each x coordinate resulted in a pressure distribution along the z-axis in which 3 droplets can be levitated. Equally, in initial acoustic field G1, the selected z coordinates resulted in a pair of nodes capable of levitating a droplet to be located near the tip of each of the capillary nozzles. To pull the 1st pair of pendant droplets, the focal points were moved downwards (i.e., away from the tip of the nozzle) along the z-axis. Acoustic field G2 in Fig. 2 was obtained by moving the focal points downward by 1 mm along the z-axis. As a result, the pair of nodes located at the tip of the nozzles moved downwards and pulled along the pendant droplets to generate a pair of levitated droplets as shown in Fig. 3 (G2). The acoustic fields G3 to G5 were obtained by moving the focal points by 1 mm for each field. This in turn moved the pair of levitated droplets downwards along the z-axis (see Fig. 3, G3 to G6) while resulting in another node capable of levitating a droplet to be located near the tip of the capillary nozzle. This implies that the total distance by which the 1st pair of levitated droplets were moved downwards along the z-axis was ∼half of the wavelength in air of sound waves emitted by the transducers. Similarly, 2nd and 3rd pairs of droplets were generated by pulling pendant droplets using acoustic forces in G7 and G12. The remaining acoustic fields from G8 to G11 and G13 in Fig. 2 were used for positioning and holding droplet pairs. The images of droplets corresponding to acoustic fields G1 to G11 are shown in Fig. 3 and Movie S1 in supplementary information.

**Fig. 4:**
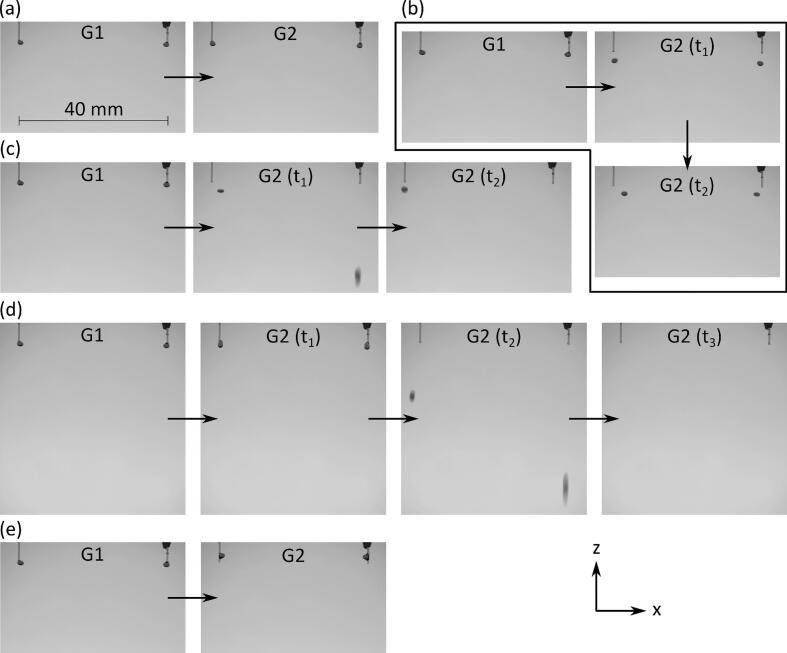
Images showing droplet generation for when the focal points were moved down by (a) 0.5 mm, (b) 1 mm, (c) 1.5 mm, (d) 2 mm and (e) 2.5 mm between G1 and G2 along the z-direction (numbers G1 and G2 corresponds to acoustic fields in Fig. 2), acoustic fields in G2(t_1_)-G2 (t_3_) is same as G2, 2 µL amaranth solution in PBS was dispensed from the pump to create each pendant droplet.

*Distance by which focal points are moved:* The distance by which the focal points were moved to pull pendant droplets determined whether the pendant droplets could be detached from the nozzle tips with the results summarized in Fig. 4 and Movies S2-S6 in the supplementary information. When the focal points were moved down by 0.5 mm, the droplets could not be detached repeatably from the tip of the capillary nozzles. In contrast, when the focal points were moved down by 2.5 mm, the droplets climbed up the walls of the nozzles as shown in Fig. 4(e). When the focal points were moved down by 1.5 and 2 mm, as shown in Fig. 4(c) and (d), one and both droplets fell after being detached from the nozzle tips, respectively. Moving the focal points down by 1 mm (see Fig. 4(b)) resulted in reliable detachment of droplets and their subsequent levitation. Thus, throughout the remainder of this work, focal points were moved by 1 mm to generate droplets.

**Fig. 5:**
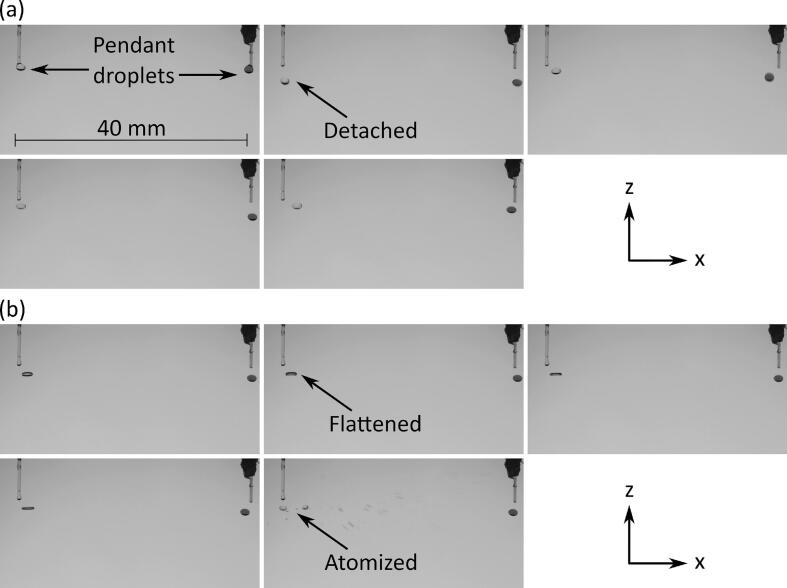
Images showing (a) generation and levitation, and (b) flattening and atomization of left-hand side droplets of 100 % DMSO while droplets on right-hand side are amaranth in aqueous PBS, 2 µL solution was dispensed to create each pendant droplet.

*Droplet composition:* The most common solvents used to perform (bio)chemical assays are aqueous buffers without and with significant concentrations of DMSO. Thus, we studied whether the approach of creating pendant droplets followed by their pull using acoustic forces can be applied to generate levitated droplets containing different ratios of water and DMSO. Movies S7-S11 in supplementary information show generation and levitation of droplets containing different percentages of DMSO in water. As shown in Movie S9, droplets containing up to 50 % DMSO in water were successfully detached from nozzles and were somewhat flattened along z direction after levitation. Droplets containing 75 % and 100 % DMSO were detached from nozzles and after levitation deformed their shapes significantly, becoming much more elongated in x direction and flattened in z direction, and often atomized as shown in Fig. 5 and Movies S10-S11. This was because the interfacial tension of air-DMSO is much lower than air–water (∼42 [26] *versus* ∼72 [27] mN/m at 298 K). Since the concentration of DMSO in any realistic assay is unlikely to exceed 5 % (v:v), this method of droplet generation is suitable for biochemical assays [28].

**Fig. 6:**
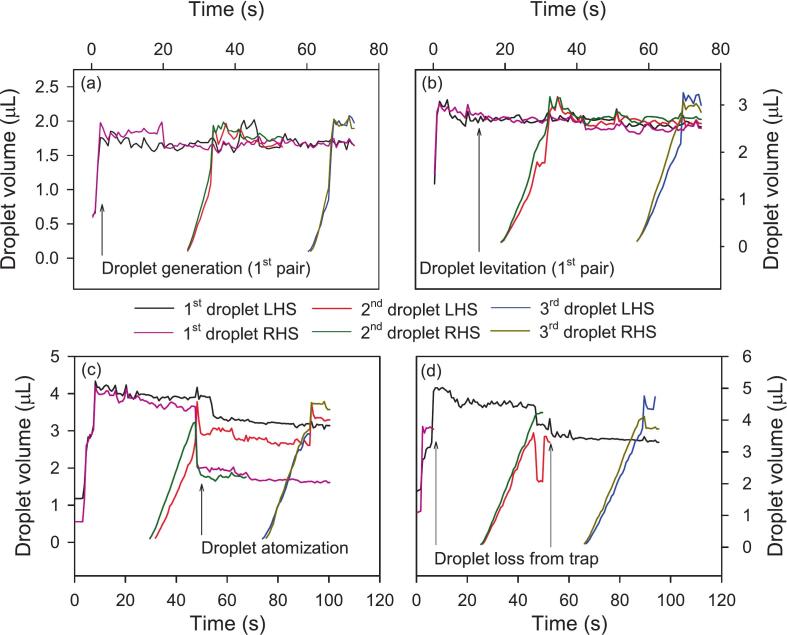
Volume of levitated droplets *versus* time for when (a) 2 µL, (b) 3 µL, (c) 4 µL and (d) 5 µL amaranth solution in PBS was dispensed to create each pendant droplet and pendant droplets were acoustically pulled to generate levitated droplets, LHS is left-hand side and RHS is right-hand side.

*Droplet volume:* The volume of generated droplets was controlled by the volume of solution dispensed using the syringe pump to create each pendant droplet. The volume of dispensed solution to create each pendant droplet varied between 1.5 and 5 µL with results of each case shown in Movies S12-S16 in supplementary information. As shown in Movie S12, 1.5 µL pendant droplets could not be repeatedly detached from nozzle tips because the capillary force exceeded the acoustic radiation force. This is because the capillary force scales with r while the acoustic force scales with r^2^ where r is the radius of the droplet [13]. This implies that as the volume and hence radius of droplets increases, acoustic force becomes greater than capillary force, allowing reliable detachment of pendant droplets to generate levitated droplets. Droplets of 2, 3, 4 and 5 µL pendant droplets were successfully detached to generate droplets as shown in Movies S13-S16 and by the rising edges in Fig. 6.

The traces in Fig. 6 were generated using ImageJ as noted in Section 2.3 to extract the major and minor axis lengths of each droplet from each video frame. In each graph, the droplets can be seen to be growing with time as the syringe pump operates, then stop growing as the droplet detaches from the capillary. The noise on the traces is a result of the droplet moving during the integration time of the camera, which tended to overestimate the size of the droplet. The droplet volume as determined using ImageJ was generally lower than the volume set on the syringe pump, which may be a result of errors in the syringe diameter or incomplete detachment of the whole droplet. It can also be seen that 4 and 5 µL droplets were prone to atomization and falling out of acoustic traps as shown in Fig. 6(c) and (d), respectively. Based on these findings, the remainder of the work was performed only on 2 and 3 µL pendant droplets.

*Peak-to-peak voltage:* Transducers were driven by 16 and 17 V peak-to-peak voltages and for each case, the number of droplets that could be generated and levitated out of a total of 60 was determined. Both 2 and 3 μL pendant droplets of amaranth solution in PBS were studied with the results summarized in Table 1. The combination of droplet volume and transducer voltage that resulted in the successful levitation of the most droplets was 2 uL and 16 V p-p, where 95 % of the droplets remained levitated for the duration of the experiment. The least successful combination was 3 uL at 16 V p-p, where only 78 % of the droplets remained levitated. The equilibrium of a levitated droplet is governed by the interplay of gravitational, acoustic, and surface tension forces. While gravitational force scales with droplet volume and acoustic force scales with both droplet size and applied voltage, it doesn’t mean that the 3 µL droplets can be stable by just increasing the voltage because exceeding the critical voltage threshold causes atomization easily. Consequently, to ensure droplet stability, reducing the step length for 3 µL droplets, for instance, to 0.5 mm in pulling procedures was found to be the most reliable method for generating droplets. The size of the droplets decreased from the top to the bottom droplet because of evaporation, as the bottom droplet was the first to be generated. 2 uL at 16 V p-p was chosen for the remaining experiments to ensure that the maximum number of droplets could be generated and remain levitated.

**Table 1:** Summary of levitated droplets generated from amaranth in PBS pendant droplets of 2 and 3 µL that were acoustically pulled when transducers are driven at 16 and 17 V peak-to-peak where L is left-hand side droplets and R is right-hand droplets.

Volume of pendant droplets (µL)	2	3	2	3
Peak-to-peak voltage (V)	16		17	
Maximum number of droplets to be generated and levitated	60			
Droplets successfully generated and levitated without atomization/ fall out of traps	57	47	54	49
Volume of top levitated droplets, *V* (µL)	L: 2.15 ± 0.12 R: 2.07 ± 0.17	L: 2.98 ± 0.20 R: 3.02 ± 0.20	L: 1.87 ± 0.16 R: 2.16 ± 0.08	L: 3.06 ± 0.40 R: 3.33 ± 0.44
Volume of middle levitated droplets, *V* (µL)	L: 1.77 ± 0.08 R: 1.72 ± 0.22	L: 2.35 ± 0.25 R: 2.55 ± 0.19	L: 1.75 ± 0.12 R: 1.82 ± 0.11	L: 2.57 ± 0.18 R: 2.53 ± 0.49
Volume of bottom levitated droplets, *V* (µL)	L: 1.77 ± 0.20 R: 1.54 ± 0.20	L: 1.80 ± 0.54 R: 2.29 ± 0.38	L: 1.65 ± 0.47 R: 1.87 ± 0.23	L: 1.97 ± 0.60 R: 2.25 ± 0.83

#### 3.1.2 Move and merge

**Fig. 7:**
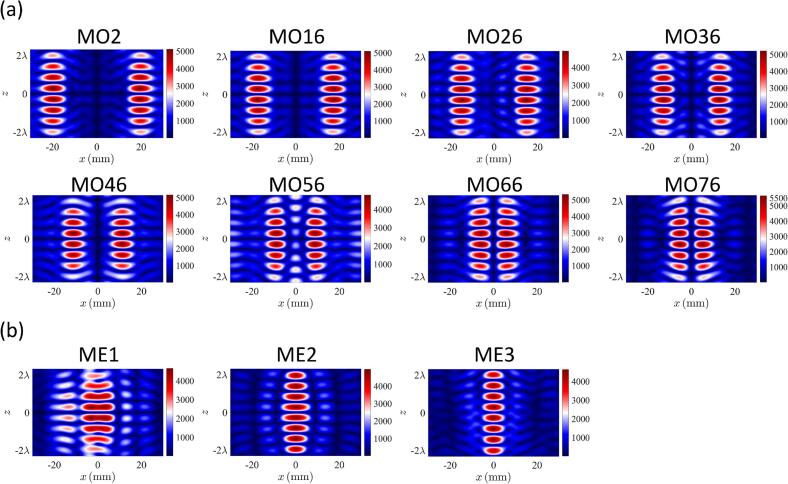
Simulated pressure distribution of the acoustic fields used to (a) move and (b) merge a pair traps.

Although move and merge of columns of levitated droplets has been reported by the authors in [11] and [1], in both these previous studies, droplets were manually pipetted in acoustic traps. Herein, we generated droplets in an automated fashion by pulling pendant droplets using acoustic forces and arranging their positions using acoustic fields G1 to G13 in Fig. 2. Subsequently, generated droplets arranged in two columns were moved towards each other and eventually merged. The two columns of droplets were brought close to each other using a sequence of 76 acoustic fields (MO1 to MO76) with the pressure distribution of the selected acoustic fields shown in Fig. 7(a). The initial separation distance along the x-axis between the two columns of traps was 40 mm. The separation distance along the x-axis between the two columns of traps was reduced by 0.4 mm. As shown in Movie S17 in supplementary information, the droplets moved closer as the acoustic traps moved closer to each other in the x-direction. However, as the distance between the two columns of traps became less than a certain value, usually 10 mm (∼wavelength of sound in air emitted by the transducers), most algorithms including DS-PAT have difficulty in finding solutions. To solve this problem, 3 acoustic fields to merge the two columns of traps were designed with their pressure distributions shown in Fig. 7(b). In acoustic field ME1, wide traps with broad pressure distribution along x-axis were used so that droplets could coalesce smoothly as shown in Movie S17 in supplementary information. However, because of the broad pressure distribution along x-axis, droplets oscillated along x-axis as shown in Movie S17 in supplementary information. Thus, to reduce the oscillation of droplets along x-axis, in acoustic field ME2 and ME3, pressure distribution along x-axis was narrowed.

### 3.2 Sequence of unit operations

**Fig. 8:**
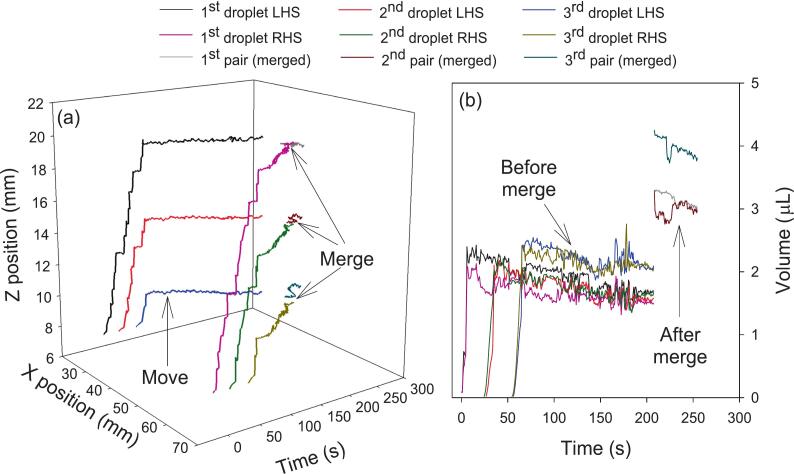
(a) X and Z positions of 3 pairs of droplets as a function of time showing the generation (represented by rising edges), move and merge and (b) volume of levitated droplets *versus* time before and after merge.

Fig. 8 shows the trajectory of three pairs of droplets and the trajectory of the three merged droplets. As noted in Section 2.3, ImageJ was used to extract the centroid coordinates of each droplet as well as its major and minor axis lengths in each frame of a video. These coordinates were then used to create a 3D plot as a function of time in Fig. 8(a), while the droplet volume was plotted as a function of time in Fig. 8(b). It can be seen that the errors in volume are much larger than the errors in centroid position. This is because the droplet oscillated rapidly (mainly in the X direction) with variable amplitude around the trap position, which meant that the droplet position was averaged out over the integration time of the camera, while the droplet size was not. Fig. 8(b) shows that the merged droplets were stable and about twice the size of the original droplets. Additionally, the droplet sizes can be seen to be decreasing slightly with time as a result of evaporation.

### 3.3 Application of the all-acoustic toolbox

After unit operations (generate, hold, move and merge) and a sequence of unit operations was realized, the all-acoustic toolbox was applied to implement an esterase enzyme assay in levitated droplets. When the enzyme esterase acts upon a colorless substrate, fluorescein diacetate (FDA), fluorescein is produced as a product [29]. FDA is almost colorless because there is no charge delocalization over the extended π structure of the molecule. In contrast, the dianionic form of fluorescein absorbs blue light of wavelength of ∼490 nm [30] and emits green light of wavelength of ∼518 nm. Furthermore, the rate of fluorescein production is proportional to the activity of the esterase enzyme.

**Fig. 9:**
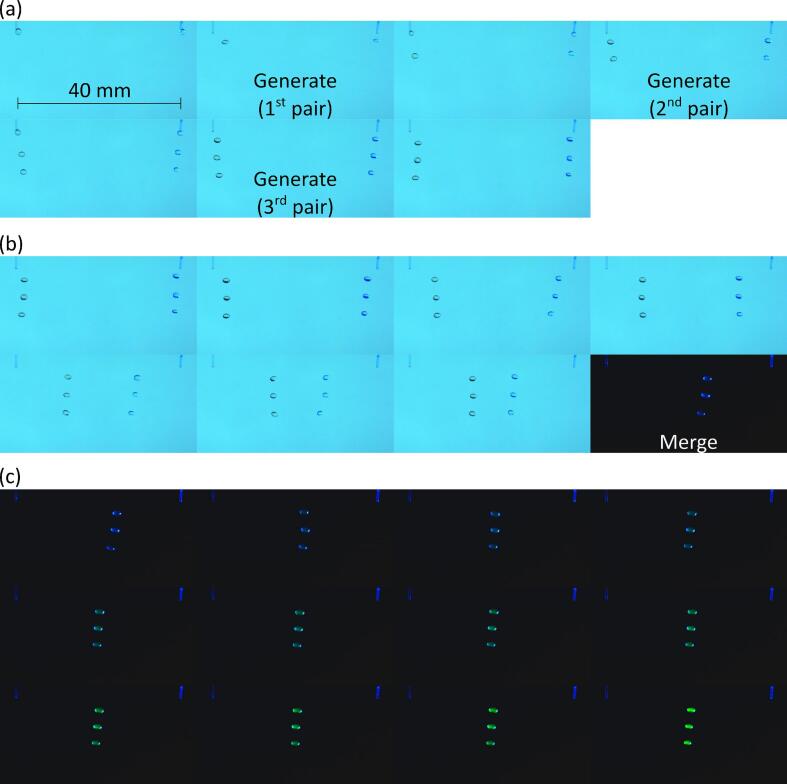
(a) Generation of 50 ppm FDA droplets (left-hand side) and 0.5 mg/mL esterase droplets (right-hand side), (b) merge and move columns of FDA and esterase droplets to start the production of fluorescein, and (c) fluorescence images of merged droplets *versus* time.

**Fig. 10:**
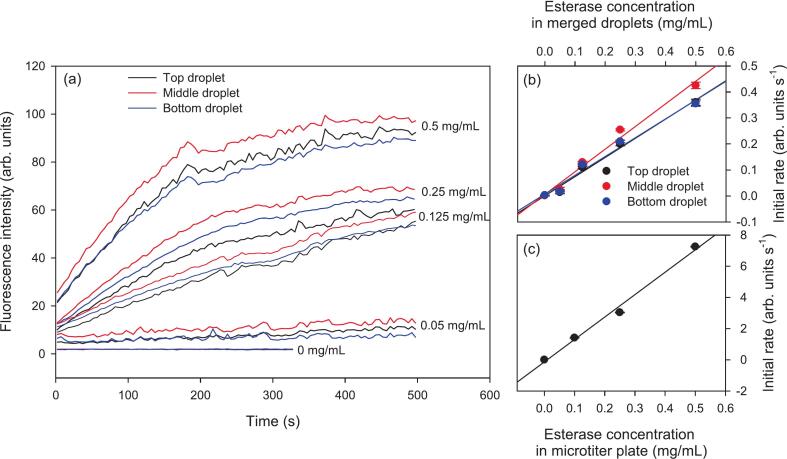
(a) Time course of the fluorescence intensity of three levitated droplets for esterase concentrations of 0, 0.05, 0.125, 0.25 and 0.5 mg/mL in merged droplets and initial rates of fluorescein *versus* esterase concentration in (b) acoustically levitated and manipulated droplets with integrated optical detection, and (c) solutions in microtiter plates with fluorescence plate reader.

To perform the esterase enzyme assay, a column of 50 ppm FDA droplets and another column of esterase droplets were generated as shown in Fig. 9(a) and Movie S18 in the supplementary information. Subsequently, the columns were moved and merged as shown in Fig. 9(b) following which, fluorescence was recorded using the integrated optical detection set-up. As shown in Fig. 9(c) and Movie S18 in supplementary information, fluorescence intensity of droplets increased with time because more fluorescein was produced as esterase acts upon FDA. The concentration of esterase in generated droplets was between 0 and 1 mg/mL. Thus, after merging with FDA droplets, the enzyme concentration in merged droplets was between 0 and 0.5 mg/mL. As shown in Fig. 10(a), the rate of fluorescein production and hence fluorescence intensity increased as the esterase concentration was changed from 0 to 0.5 mg/mL in merged droplets.

The fluorescence intensity data in Fig. 10(a) for the first 50 s for each enzyme concentration was fitted to a straight line to determine the initial rate. The relationship between the initial rate and esterase concentration in merged droplets was linear as shown in Fig. 10(b), which is in line with the results obtained by performing the same assay in microtiter plates and fluorescence plate reader (see Fig. 10(c)). The initial rates in the two cases cannot be compared quantitatively because the optical path lengths through which fluorescence signal was recorded were different in case of levitated droplets and solutions in wells of microtiter plates. Six runs with three droplets per run of 0.5 mg/mL esterase in merged droplets were carried out. Initial rates were generated using fluorescence intensity data recorded over the first 50 s fitted to straight lines and time constants were generated by fitting the fluorescence intensity recorded for 500 s to exponential rise to maximum of the form F = F_0_ + a(1-exp(−b × t)) where F_0_, a and b were constants determined by fitting the data, F is fluorescence intensity and t is time.

**Fig. 11:**
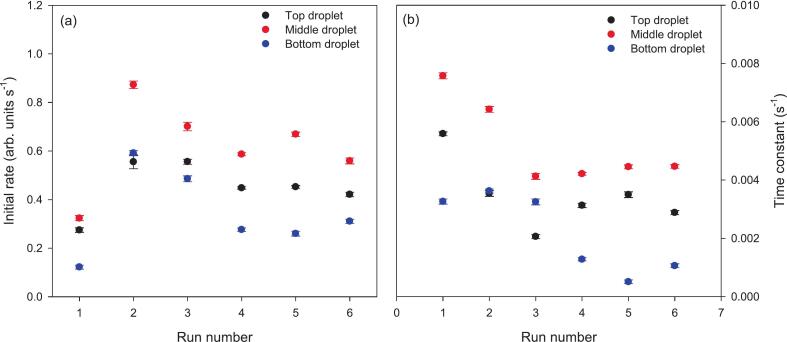
(a) Initial rates of fluorescein production over the first 50 s after droplet merging for esterase concentration 0.5 mg/mL in merged droplets and six repeats, and (b) time constants derived from a fit to an exponential rise to a maximum for fluorescein production for esterase concentration 0.5 mg/mL and six repeats.

A summary of the initial rates and the time constants of the esterase assay in top, middle and bottom droplets for the 6 repeats at 0.5 mg/mL esterase concentration in merged droplets is presented in Fig. 11. Average initial rates and time constants including standard deviations for top, middle and bottom droplets for the 6 repeats at 0.5 mg/mL esterase concentration in merged droplets are summarized in Table 2. It can be seen that there is a large variation in both the initial rates and time constants, but the middle droplet always has the highest initial rate and time constant. This is consistent with our previous findings [11], where the reaction was fastest in the trap with the highest acoustic forces. Our working hypothesis is that higher the acoustic forces result in greater acoustic streaming and thus more stirring and faster reaction rates. The middle droplets may experience the largest acoustic pressure compared with top and bottom droplets. This would also explain why the time constants in levitated droplets are higher than in unstirred microtiter plates. However, the acoustic field should be uniform along the z axis according to our design in Fig. 7(b) ME3. This can be attributed to imperfections in the transducer elements, since the design was predicated on an idealized model that does not account for inter-element variations. And it can also be verified from our previous work [1] where the experimental results showed nonuniformity nevertheless the design is uniform along the z axis.

**Table 2:** Average initial rate and exponential fit time constant for top, middle and bottom droplets with 95 % confidence limits for esterase concentration 0.5 mg/mL in merged droplets and six measurements, and the corresponding time constant for an assay performed in solutions in a microtiter plate.

Position of droplet	Initial rate (arb. units s^−1^)	Time constant (s^−1^)	
		Droplets	Microtiter plate (run 1, run 2)
Top	0.45 ± 0.11	3.45 × 10^−3^ ± 1.24 × 10^−3^	2.1 × 10^−3^ ± 8.44 × 10^−6^, 1.9 × 10^−3^ ± 6.55 × 10^−6^
Middle	0.62 ± 0.19	5.21 × 10^−3^ ± 1.51 × 10^−3^	
Bottom	0.34 ± 0.18	2.17 × 10^−3^ ± 1.43 × 10^−3^	

Although the DS-PAT algorithm attempts to find a solution with the smallest pressure differences between traps, it is clear from the simulations that the trap pressures are not exactly equal. The variation in pressures, and hence reaction rates, is smaller than in our previous work using a simpler algorithm where the pressure differences were much greater. It may be necessary to measure the trap pressures and feed this back into the DS-PAT algorithm to ensure the trap pressures are equal.

## 4 Conclusions

We have designed an all-acoustic toolbox based on phased arrays, pumps and capillary nozzles with the algorithm DS-PAT and presented series of operations including generation, hold, move and merge of droplets. Droplets of volume between 1.5 and 5 µL could be generated, but the smallest droplet could not be consistently pulled from the dispensing capillary, while the largest droplets tended to fall from the acoustic traps or to atomize. Optimization showed that 95 % of 2 µL droplets generated and trapped using 16 V p-p transducer voltage could be held for more than 200 s. The error in volume of the generated droplets varied from 5.6 % for 2 µL droplets at 16 V p-p to 13 % for 3 µL droplets at 17 V p-p. For 2 µL droplets at 16 V p-p this is better than the ISO 8655–2 standard for manual pipettes. It was possible to generate and hold two columns of three droplets by using sequential dispensing and pulling from the capillary.

After optimization of droplet generation, moving and merging of columns of droplets was carried out to show that the volumes of the merged droplets were consistent with the sum of the individual droplets and that the larger merged droplet would remain levitated without falling from the trap or atomizing.

Finally, the sequence of droplet generation, moving, merging and holding was used to carry out an enzyme assay using esterase and fluorescein diacetate. As found in our previous work, the reaction rate was dependent on the droplet position in the acoustic traps. The middle droplet consistently reacted faster than the top or bottom droplets in a column of three droplets, although the difference in reaction rates was smaller than in our previous work. We hypothesize that this is a result of higher acoustic pressure in the middle droplet resulting in faster acoustic streaming in the droplet and thus more effective stirring and faster reaction rates. Use of the DS-PAT algorithm resulted in more uniform acoustic pressures and hence less variation in the reaction rates compared to our previous work using less effective algorithms [11].

We have demonstrated the automated unit operations of droplet generation, moving, merging and optical detection and used these to show that it is possible to completely automate a biochemical assay using multiple acoustically levitated droplets. As far as we are aware, this is the first time this has been shown where multiple assays (three in this case) have been carried out simultaneously in levitated droplets. Future work will increase the number of droplets that can be generated to permit more complex assays requiring multiple sequential additions.

## CRediT authorship contribution statement

**Jianqing Li:** Writing – review & editing, Writing – original draft, Visualization, Validation, Software, Investigation, Formal analysis, Data curation. **Nicholas J. Goddard:** Writing – review & editing, Writing – original draft, Visualization, Validation, Software, Methodology, Formal analysis, Conceptualization. **Khalid Haliru:** Writing – review & editing, Investigation. **Ruchi Gupta:** Writing – review & editing, Writing – original draft, Visualization, Validation, Supervision, Resources, Project administration, Methodology, Funding acquisition, Formal analysis, Data curation, Conceptualization.

## Declaration of competing interest

The authors declare that they have no known competing financial interests or personal relationships that could have appeared to influence the work reported in this paper.
